# Effect of Quercetin on Injury to Indomethacin-Treated Human Embryonic Kidney 293 Cells

**DOI:** 10.3390/life11111134

**Published:** 2021-10-25

**Authors:** Chun Chen, Jai-Sing Yang, Chi-Cheng Lu, Yu-Tse Wu, Fu-An Chen

**Affiliations:** 1 School of Pharmacy, College of Pharmacy, Kaohsiung Medical University, Kaohsiung City 807, Taiwan; u106830006@kmu.edu.tw; 2 Department of Medical Research, China Medical University Hospital, China Medical University, Taichung 404, Taiwan; jaisingyang@gmail.com; 3 Department of Sport Performance, National Taiwan University of Sport, Taichung 404, Taiwan; a722353@ntupes.edu.tw; 4 Department of Pharmacy and Master Program, Tajen University, Pingtung 907, Taiwan

**Keywords:** apoptosis, antioxidant, human embryonic kidney 293 (HEK293) cells, indomethacin, quercetin, mitochondrial membrane potential (ΔΨm)

## Abstract

Nonsteroidal anti-inflammatory drugs (NSAIDs) are used to treat inflammation and pain and even to prevent the progression of cardiovascular disease. They have become widely used because of their effectiveness, especially among athletes performing high-intensity training. Indomethacin is used for pain management in sports medicine and is highly effective and versatile. However, several clinical studies have reported that indomethacin induces acute renal damage. In the present study, we determined that indomethacin reduced human embryonic kidney 293 (HEK293) cell viability in a concentration-dependent manner by triggering apoptosis. In addition, we demonstrated the effect of quercetin on indomethacin-treated HEK293 cells by inactivating the caspase-3 and caspase-9 signals. Furthermore, quercetin reduced ROS production and increased mitochondrial membrane potential (ΔΨm) in indomethacin-treated HEK293 cells. Our results indicate that quercetin can interrupt the activated caspase and mitochondrial pathway induced by indomethacin in HEK293 cells and affect apoptotic mRNA expression. Quercetin can protect against indomethacin-induced HEK293 cell apoptosis by regulating abnormal ΔΨm and apoptotic mRNA expression.

## 1. Introduction

Indomethacin, a nonsteroidal anti-inflammatory drug (NSAID), is a powerful prostaglandin synthesis inhibitor [1]. Its effectiveness has made it popular for pain relief [2,3] and for the prevention of pancreatitis after endoscopic retrograde cholangiopancreatography; indomethacin is also used for pain reduction in delayed-onset muscle soreness and for clinical treatment of rheumatoid arthritis [4,5,6,7]. However, researchers have reported adverse reactions to indomethacin, such as fluid retention, blood clots, myocardial infarction, hypertension, ulceration of the stomach or intestine, and impaired renal function [8,9,10,11,12,13].

Through regular, high-intensity training, athletes can easily experience sports injuries and are likely to use medications and supplements [14,15]. Athletes also usually experience muscle fatigue and fasciitis caused by long-term training. Furthermore, daily NSAID use is prevalent despite package inserts cautioning against chronic NSAID use [16,17]. No particular restrictions on nonsteroidal analgesics or anti-inflammatory drugs have been implemented. Therefore, painkillers and anti-inflammatory drugs are commonly used, and even abused, for treating sports injuries [18,19].

Excessive exercise increases myoglobin and creatine kinase, which can induce exercise-induced rhabdomyolysis and even acute renal failure [20,21,22]. Indomethacin reduces blood flow to the kidneys; such reduced blood flow easily changes the permeability of the glomerular basement membrane and causes acute damage to the kidney [13]. During exercise, renal hemodynamics, including electrolyte and protein excretion, may cause renal blood flow to decrease to one-quarter of the normal level [23]. Because of the changes in renal function during exercise, indomethacin intake can compromise kidney function and increase acute renal failure incidence [13].

NSAIDs are commonly consumed as a fever reducers and analgesics, and because of their versatile effects, their daily use has become common [24]. Because athletes frequently experience physical stress and injuries, they might use painkillers to relieve discomfort [25]. Nevertheless, the adverse consequences and side effects of most of these drugs are underestimated [24,25]. NSAID intake can increase the chronic kidney disease (CKD) risk, and this risk may be greater than previously estimated. In a retrospective study of American military servicepeople, those using NSAIDs had 20% greater risks of acute kidney injury (AKI) and CKD [24]. However, although basic NSAIDs are versatile medications that are available over the counter (OTC), NSAIDs can induce adverse drug reactions that can result in hospitalization and death [26].

Flavonoids have been reported to be potential antioxidant chemopreventive agents [27,28,29]. In particular, quercetin has shown protective in vitro and in vivo abilities against chemically induced acute kidney damage by inducing antioxidant, inflammatory, and glomerular ultrastructural effects [30,31,32,33,34].

Indomethacin can reduce glomerular filtration and renal blood flow [13]. In the present study, we investigated the indomethacin-induced mitochondrial membrane potential (ΔΨm). Indomethacin-induced apoptosis involves the intrinsic apoptosis pathway and altered expression of caspase family proteins. To reduce or prevent adverse reactions and guide safe medication use in clinical settings, we investigated the pathway of indomethacin to understand how indomethacin induces kidney injury. In addition, we investigated quercetin as a potential preventive agent against indomethacin-induced kidney mitochondrial malfunction.

## 2. Results

### 2.1. Effect of Quercetin on Viability of Human Embryonic Kidney 293 Cells Treated with Indomethacin

**Figure 1:**
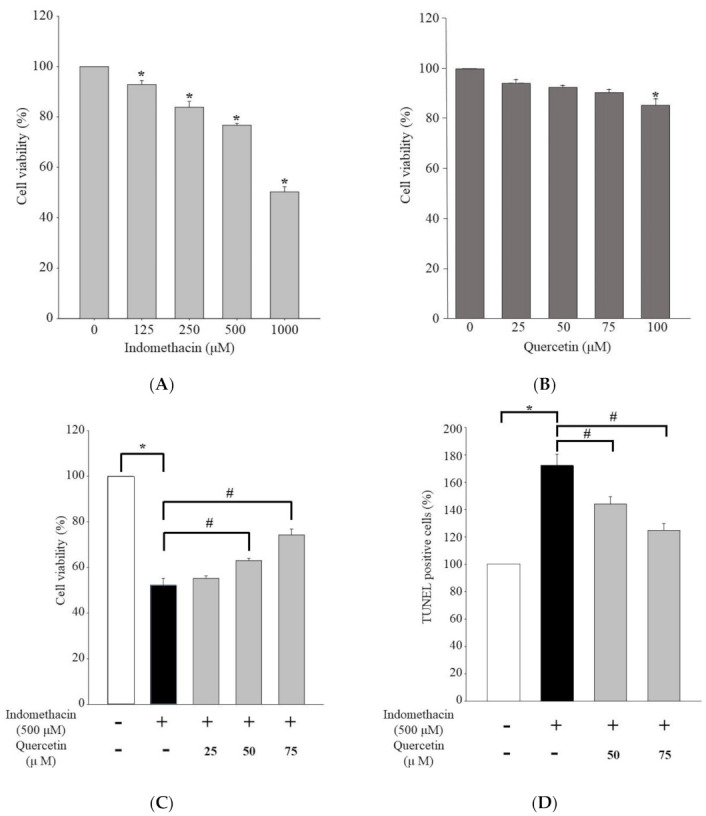
Viability of HEK293 cells treated with (**A**) indomethacin (0, 125, 250, 500, or 1000 μM) or (**B**) quercetin (0, 25, 50, 75, or 100 μM). (**C**) Viability of indomethacin−treated HEK293 cells exposed to 25, 50, or 75 μM quercetin. (**D**) TUNEL−positive cells without quercetin or treated with 50 or 75 μM quercetin and 500 μM indomethacin were considered apoptotic. Values are expressed as means ± standard errors (n = 8). Dunnett’s test was used to identify significant differences (* *p* < 0.05 vs. untreated cells; # *p* < 0.05 vs. cells with indomethacin treatment).

Figure 1 presents the viability results for human embryonic kidney 293 (HEK293) cells after treatment with indomethacin at 0, 125, 250, 500, or 1000 μM. The viability of the HEK293 cells treated with indomethacin was significantly lower at higher indomethacin concentrations (Figure 1A).

We treated the HEK293 cells with 0, 25, 50, 75, or 100 μM quercetin (Figure 1B). At 25, 50, and 75 μM, quercetin increased the viability of the HEK293 cells treated with indomethacin (Figure 1C). In particular, the cells treated with 50 and 75 μM quercetin exhibited significant differences from the untreated cells. The trend in apoptotic cells was the reverse; quercetin treatment protected against apoptosis in indomethacin-treated cells in a concentration-dependent manner, as determined through transferase-mediated d-UTP nick end labeling (TUNEL) assay (Figure 1D, Table S1). Therefore, quercetin can protect against indomethacin-induced renal injury.

### 2.2. Effect of Quercetin on Caspase-Dependent Apoptosis in HEK293 Cells Treated with Indomethacin

**Figure 2:**
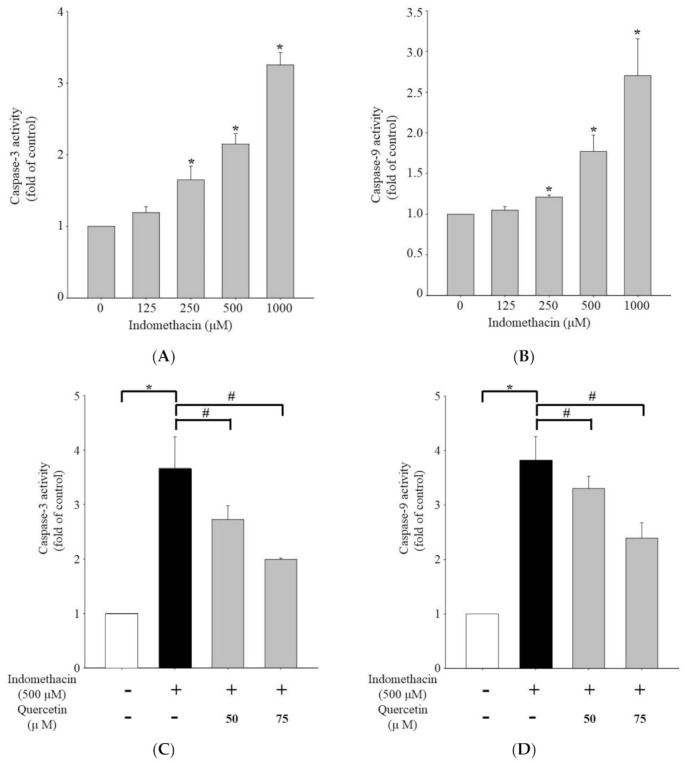
Effect of indomethacin on caspase activity in HEK293 cells. Fold changes in (**A**) caspase-3 and (**B**) caspase-9 activity in HEK293 cells after incubation with 125, 250, 500, or 1000 μM indomethacin for 24 h (vs. 0 μM). (**C**) Caspase−3 and (**D**) caspase−9 activity in indomethacin−treated HEK293 cells in the presence of 50 or 75 μM quercetin. Values are expressed as means ± standard errors (n = 8). Dunnett’s test was used to determine significant differences (* *p* < 0.05 vs. untreated cells; # *p* < 0.05 vs. indomethacin treatment only).

To identify the intrinsic apoptosis pathway triggered by indomethacin in HEK293 cells, we measured the fold change in caspase levels versus the control cells. The addition of indomethacin significantly increased caspase-3 protein in HEK293 cells compared with the untreated cells (Figure 2A). Therefore, the caspase cascade pathway is associated with the mechanism through which indomethacin induces apoptosis in HEK293 cells. An increase in caspase-9 dependent on the indomethacin concentration occurred in the HEK293 cells, suggesting that indomethacin-induced apoptosis is related to increases in caspase-3 and caspase-9 levels (Figure 2B). Quercetin at 50 and 75 μM resulted in significantly lower caspase-3 activity than was observed in the cells treated with indomethacin alone (Figure 2C). A similar trend in caspase-9 activity occurred (Figure 2D, Table S1). Therefore, quercetin treatment reduces the increased caspase-3 and caspase-9 activity in indomethacin-treated HEK293 cells.

### 2.3. Effects of Quercetin on Superoxide Anion, Mitochondrial Superoxide, and Reactive Oxygen Species Production in HEK293 Cells Treated with Indomethacin

**Figure 3:**
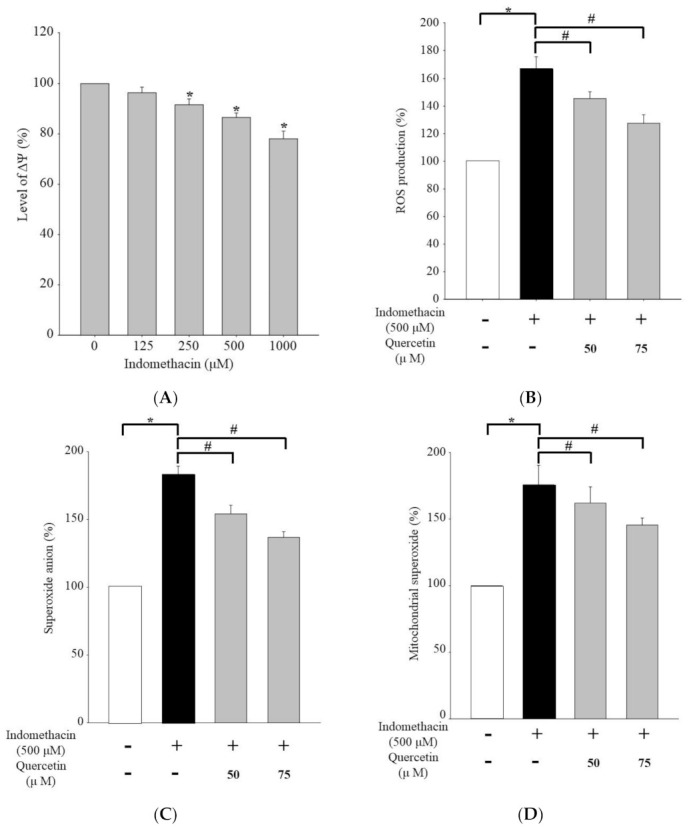
(**A**) Results for ΔΨm in HEK293 cells incubated with indomethacin (0, 125, 250, 500, or 1000 μM) for 24 h. (**B**) ROS production results in HEK293 cells treated with 500 μM indomethacin and 50 or 75 μM quercetin. (**C**) Superoxide anion production results in HEK293 cells treated with 50 or 75 μM quercetin and 500 μM indomethacin. (**D**) Mitochondrial superoxide levels in HEK293 cells treated with 50 or 75 μM quercetin and 500 μM indomethacin. Indomethacin treatment was confirmed using DiOC6(3) fluorescent dye in flow cytometry analysis. ROS levels, superoxide anion production, and mitochondrial superoxide were respectively assessed using H_2_DCFDA, dihydroethidium, and MitoSOX fluorescent dye staining in flow cytometry. Values are expressed as means ± standard errors (n = 8). Dunnett’s test was used to determine significant differences (* *p* < 0.05 vs. untreated cells; # *p* < 0.05 vs. indomethacin treatment only).

We demonstrated that indomethacin-induced apoptosis involves an active caspase-3/9-dependent pathway; in addition, quercetin can play a crucial role in suppressing caspase-dependent protein expression. To determine the effects of indomethacin and quercetin on upstream molecular signaling in HEK293 cells, we measured reactive oxygen species (ROS) production and mitochondrial membrane potential, as shown in Figure 3. To investigate indomethacin-induced apoptosis, a flow cytometric assay was used to determine ΔΨm, which was significantly lower in the HEK293 cells treated with higher concentrations of indomethacin (*p* < 0.05; Figure 3A), indicating that indomethacin triggers mitochondrial malfunction. We also detected ROS production; in indomethacin-treated cells, the ROS level significantly increased; however, quercetin cotreatment resulted in significant decrease in the indomethacin-induced elevated ROS production (Figure 3B). Similar results were obtained for superoxide anion and mitochondrial superoxide levels (Figure 3C,D). Indomethacin significantly induced ROS production, explaining its ability to cause mitochondrial malfunction and subsequent apoptosis. In addition, quercetin exposure exerted an ostensible antioxidant effect on indomethacin-reduced peroxidation.

### 2.4. Effects of Pan-Caspase Inhibitor Z-VAD-FMK on Apoptosis in HEK293 Cells Treated with Indomethacin

**Figure 4:**
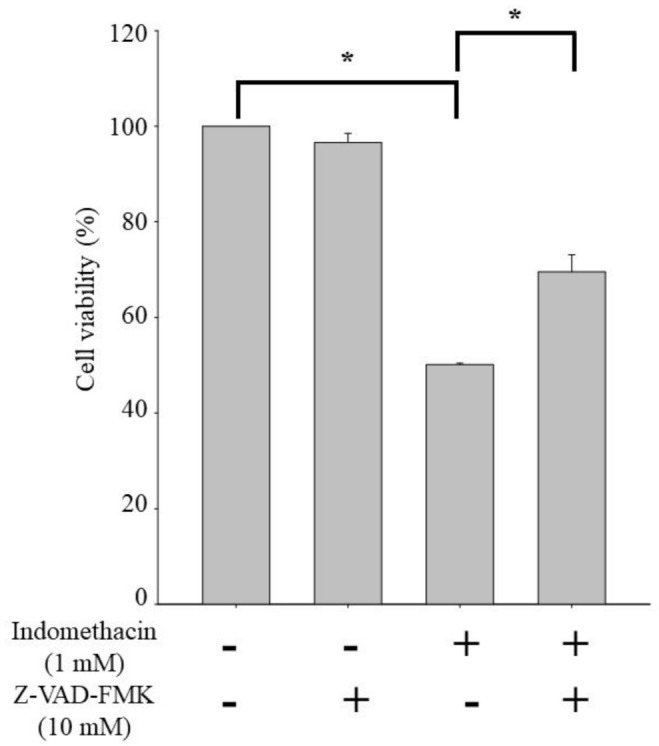
Apoptosis in indomethacin−treated HEK293 cells treated or untreated with the pan-caspase inhibitor Z−VAD−FMK. Treated cells were exposed to 10 μM Z−VAD−FMK for 24 h before indomethacin treatment. Values are expressed as means ± standard errors (n = 8). Dunnett’s test was used to determine significant differences (* *p* < 0.05 vs. untreated cells; * *p* < 0.05 vs. indomethacin treatment only).

To show how protein expression regulates apoptosis in indomethacin-treated HEK293 cells, we used Z-VAD-FMK, which is a pan-caspase inhibitor, to block caspase-3 and caspase-9 activity. After exposure to Z-VAD-FMK, apoptosis in the indomethacin-treated and untreated HEK293 cells did not differ significantly (Figure 4).

### 2.5. Effects of Quercetin on Apoptotic mRNA Expression in HEK293 Cells Treated with Indomethacin

**Figure 5:**
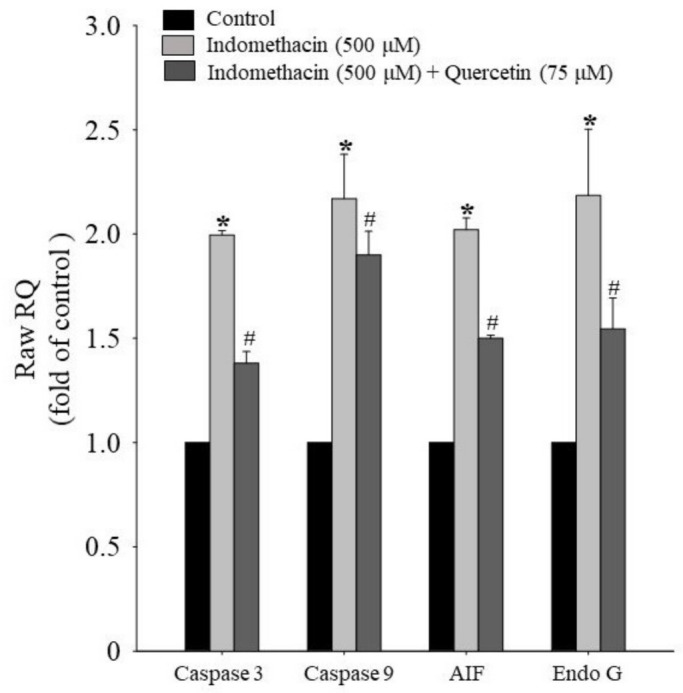
Effects of quercetin on apoptotic mRNA expression in HEK293 cells treated with indomethacin (500 μM) and with or without quercetin (75 μM) for 24 h. Total mRNA was extracted to determine expression of caspase-3, caspase-9, AIF, and EndoG mRNA. Values are expressed as means ± standard errors (n = 3). Dunnett’s test was used to determine significant differences (* *p* < 0.05 vs. untreated cells; # *p* < 0.05 vs. indomethacin treatment only).

To identify the caspase-dependent pathway and the effect of mRNA expression in indomethacin-treated HEK293 cells with or without quercetin cotreatment, we used caspase-3, caspase-9, AIF, and EndoG mRNA expression to represent mitochondrial function. We determined that quercetin has the potential to reduce apoptotic mRNA expression, including that of caspase-3, caspase-9, AIF, and EndoG (Figure 5).

## 3. Discussion

NSAIDs contribute to AKI by reducing renal blood flow, which induces renal tubule obstruction through crystal deposition; NSAIDs causes cytotoxicity to renal cells, cell-mediated immune impairment, and acute interstitial nephritis [35]. CKD incidence was observed to be greater in patients who were administered NSAIDs [36]. In addition, although NSAIDs pose a risk of adverse drug reactions, they are still commonly used by patients with CKD [37]. Therefore, improving risk for patients with CKD is clinically desirable [26,38,39,40]. AKI is growing in global prevalence, resulting in significant substantial mortality and morbidity in which is requiring considerable medical resources. NSAIDs and various other nephrotoxic drugs are crucial causes of AKI, while exposure to such drugs is influenced by factors such as CKD status and age [41]. Older patients with renal disease are more likely than the general population to develop AKI from NSAID exposure; however, using evidence of the renal harm due to NSAIDs on complex interventions may be effective in reducing inappropriate NSAID prescriptions for high-risk populations [42,43]. Other studies have provided evidence for clinicians to use to minimize the risk of AKI when administering NSAIDs [26,41,44]. Approximately 13% to 18% of older adults and patients in hospitals develop end-stage renal disease; furthermore, older adults using NSAIDs at high doses have a 26% greater risk of acute renal failure. People with moderate to severe kidney disease who regularly use OTC NSAIDs typically also have NSAID prescriptions [36,45,46,47]. In clinical practice, commonly used NSAIDs such as aspirin and indomethacin can cause various forms of kidney damage, including acute interstitial nephritis, acute tubular necrosis, chronic renal tubular interstitial disease, renal tubular acidosis, and glomerular disease [23,48,49,50,51,52].

On the other hand, cyclooxygenase (COX) is the enzyme, involved in oxidizing arachidonic acid (AA), also known as the index of information and pain. COX contributes to inflammation processes in organs. The development, perfusion, water handling, and renin release in the kidney can be regulated by COX-2 in a normal or paraphysiological situation. Thus, patients are facing the risk of renal ischemia on NSAIDs, which reduce vasodilatory prostaglandins synthesis [53,54,55]. COX-2 plays a metabolites regulator in the kidney, in addition, NSAIDs long-term used could imbalance the renal homeostasis of COXs and bring the risk of renal injury [56,57].

Indomethacin induces renal epithelial cell injury by downregulating the Akt- and STAT-3-related pathways [58,59]. In the present study, indomethacin upregulated caspase pathways. Specifically, indomethacin increased caspase-3 and -9 expression to induce apoptosis (Figure 2A,B and Figure 4). By contrast, cotreatment with quercetin resulted in significantly lower caspase-3/-9 activity in indomethacin-treated cells (Figure 2C,D). Mitochondria depend on the apoptosis pathway and caspase-3 and -9 for morphological changes, and ROS cleaving can indicate mitochondrial malfunction and result in insufficient energy reserves and intracellular signaling pathway activation [60,61]. In this study, we demonstrated the potential of quercetin to counteract indomethacin-induced caspase overexpression.

Long-term use of NSAIDs could cause a specific form of kidney disease which is characterized by papillary necrosis and interstitial scarring. COX2 inhibition may contribute to injury of the renal medulla [62]. In rat renal papilla cells, indomethacin (1 μg/mL) produced large inhibition of prostaglandin output from the renal papilla which provided the potential for renal injury [63]. Previous study has demonstrated indomethacin showed toxicity starting from concentrations of 10 μM to 100 μM in HEK293T cells in a dose-dependent manner [64]. Indomethacin also provided the promotion of extracellular signal-regulated kinase (ERK), p38 MAPK, and c-Jun N-terminal kinase (JNK) in 786-O renal cell carcinoma cells [59].

However, HEK293 cells have their own limitations on application. Researchers have discovered HEK293 cells have an unexpected relationship of neurons and begun to recognize which cannot be used as a normal model for renal function. In this study, we prefer to demonstrate the feasible potential of indomethacin and quercetin combo in cell lines. Accordingly, we selected HEK293 cells as an experimental model [65,66,67]. The toxicity of active drug components causes various types of kidney damage including cytotoxic damage to kidney mitochondrial and organelles; direct damage to the structure and function of mitochondria or mitochondrial death can lead to cell apoptosis. Mitochondrial apoptosis crucially regulates cell and death [68,69]. Indomethacin has been reported to instigate the endoplasmic reticulum (ER) stress response and ER Ca^2+^ mobilization, both of which increase oxidative stress and induce mitochondrial dysfunction, regardless of oxygenation conditions [70,71,72]. We investigated the oxidation conditions and ΔΨm in HEK293 cells. Indomethacin reduced ΔΨm levels by inducing mitochondrial malfunction; conversely, with quercetin cotreatment, the level exhibited a significant reduction (Figure 3A). Mitochondrial membrane permeability is enhanced by the inhibition of ADP/ATP transportase in the mitochondrial membrane, causing mitochondrial disruption, or by the induction of calcium release in the ER and intracellular calcium flow, causing the ER stress reaction and activating phospholipase A2 for renal tubular epithelial cell apoptosis and toxicity [73]. Quercetin exposure ostensibly increased antioxidant activity in cells with indomethacin-reduced peroxidation (Figure 3B–D). Quercetin can reduce oxidation levels in renal cells to inhibit the macrophage chemotaxis induced by ferroptosis in AKI and balance macrophage polarization [74,75]. Oxidative stress is a mechanism through which various drugs cause kidney damage. Quercetin can reverse ΔΨm imbalance because of its antioxidant and free radical scavenging abilities [27,76]. In the clinic, the maximum daily recommended dose of indomethacin is 200 mg. The cell exposure in this study is higher than the situation of forecast from oral administration pharmacokinetics in the maximum daily dose (MDD) [77]. For studying the preventive effect of quercetin from indomethacin-induced injury we selected a higher concentration than the maximum daily dose. We demonstrated that quercetin has significant protection even in high concentration indomethacin treated (500 μM) situation in HEK293 cells.

Mitochondria depend on apoptosis pathways that involve ROS production, DNA damage, ΔΨm imbalance, and the release of the signal transporters Apaf-1, cytochrome c, procaspase-9, and AIF [78,79,80]. When ROSs induce DNA damage, the kinase that acts upstream of p53 autophosphorylation ataxia-telangiectasia mutates; after serial phosphorylation, downstream apoptotic factors such as Fas, CD95, DR4, DR5, and TNFR are expressed to induce apoptosis [81,82]. When ROS-induced DNA damage occurs, active autophagy in the cell continues, inducing the release of a DNase from mitochondria [83,84,85,86]. We demonstrated that with quercetin exposure, the level of apoptosis- and autophagy-related factors was lower in indomethacin-treated HEK-293 cells (Figure 5). *AIF* and *EndoG* are redox sensors in the metabolic pathways for apoptosis [83,87]. In the experiment, we found that quercetin had the potential to regulate ROS production and apoptosis- and autophagy-related factors.

The adverse consequences and side effects of NSAIDs are often underestimated [24,25]. They pose a high risk of AKI in people who use them without professional consultation, including athletes and older adults, as well as in long-term users [15,16,26,88]. In conclusion, indomethacin poses a substantial risk of renal damage; the present findings that indomethacin increased ROS production and ΔΨm imbalance and upregulated caspase-3 and -9 as well as caspase-3/-9, *AIF,* and *EndoG* mRNA expression indicate that indomethacin induces renal injury. Knowledge of the risks of NSAID use can reduce the incidence of nephrotoxicity. Traditional and complementary medicine has grown in popularity because of its natural appeal and availability. Thus, research has targeted safe ingredients from Chinese medicine for use as complementary medicine to reduce the side effects of the pain and anti-inflammatory drugs taken by athletes, older adults, and patients on long-term pain medication [29,89,90]. Quercetin has therapeutic potential in cases of renal damage [91,92]. Previous studies have concluded that quercetin may effectively treat renal injury caused by chemical compounds that induce hypoxia [30,32,93,94]. In the present study, we used quercetin as a natural agent to reduce indomethacin-induced renal injury.

## 4. Materials and Methods

### 4.1. Materials

We obtained an In Situ Cell Death Detection Kit (Fluorescein), thiazolyl blue tetrazolium bromide (MTT), indomethacin, quercetin, Dulbecco’s modified Eagle’s medium (DMEM), DNase, and other reagents and chemicals from Sigma–Aldrich, Merck KGaA (Darmstadt, Germany). We obtained Muse Caspase-3/9 Assay Kits and the pan-caspase inhibitor Z-VAD-FMK from Millipore, Merck KGaA (Darmstadt, Germany). We obtained dihydroethidium, 2′,7′-dichlorodihydrofluorescein diacetate (H_2_DCFDA), 3,3′-dihexyloxacarbocyanine iodide [DiOC6(3)], and MitoSOX from Molecular Probes, Thermo Fisher Scientific (Waltham, MA, USA). We purchased L-glutamine, fetal bovine serum (FBS), trypsin-EDTA, and penicillin/streptomycin from HyClone, GE Healthcare Life Sciences (Logan, UT, USA).

### 4.2. Cell Culture

We purchased HEK293 cells from the American Type Culture Collection (Manassas, VA, USA). We cultured the cells in DMEM with 20% FBS, 2 mM L-glutamine, and antibiotics (penicillin/streptomycin) in a 5% CO_2_ humidified atmosphere at 37 °C until 100% confluence was reached; we replaced the medium every 48–72 h. The cells were collected for cell viability testing; ROS, superoxide anion, and mitochondrial superoxide production assays; caspase-3 and -9 assays, and TUNEL assay.

### 4.3. Cell Viability Assay

The HEK293 cells (2.5 × 10^4^ cells/well) were placed into 96-well plates with quercetin (0, 25, 50, 75, or 100 μM) and subsequently exposed to indomethacin at concentrations of 125, 250, 500, or 1000 μM for 24 h to induce apoptosis before 1 h with or without treatment with 10 μM Z-VAD-FMK; the cells were subsequently subjected to 2 h of treatment with 0.5 mg/mL MTT solution. Finally, 100 μL of dimethyl sulfoxide was added to the wells to dissolve formazan crystals and replace the culture medium. The optical density was measured at 570 nm by using a spectrophotometer as previously described [29,95].

### 4.4. TUNEL Assay for Apoptosis Analysis

With 50 or 75 μM quercetin, 2.5 × 10^5^ cells/mL were plated into 24-well plates and treated with 500 μM indomethacin for 24 h. Before harvest, the cells were rinsed in phosphate-buffered saline. To detect apoptosis, flow cytometry was performed using a BD FACSCalibur Flow Cytometer (BD Biosciences, San Jose, CA, USA), and the In Situ Cell Death Detection Kit (Fluorescein; Sigma–Aldrich, Merck KGaA) was used in accordance with manufacturer instructions to stain the cells. BD Cell Quest Pro Software version 5.1 (BD Biosciences) was used in accordance with a previously described procedure [96] to quantify the cells testing positive in the TUNEL assay.

### 4.5. Determination of Caspase-3 and -9 Activity

Cells (1 × 10^6^ cells/mL) were cultured in a 10-cm dish with 50 or 75 μM quercetin and then treated with 500 μM indomethacin for 24 h for the measurement of caspase protein expression. Before being harvested through centrifugation (400× *g*; Caspase-3 and Caspase-9 Colorimetric Assay Kits, R&D Systems Inc., Minneapolis, MN, USA), the cells were cultured in the working solution from the Muse Caspase-3/9 Assay Kits (Millipore; Merck KGaA) in accordance with manufacturer protocols [95,97].

### 4.6. Determination of ROS and Mitochondrial Superoxide Production through Flow Cytometry

HEK293 cells (2.5 × 10^5^ cells/mL) were cultured with 50 or 75 μM quercetin and then treated with 500 μM indomethacin for 24 h. They were subsequently centrifuged for 5 min at 400× *g*. The cell pellets were harvested and suspended in a 500-μL staining solution of 10 μM H2DCF-DA, dihydroethidium, or MitoSOX for ROS, superoxide anion, or mitochondrial superoxide detection, respectively, and subsequently incubated for 30 min at 37 °C. ROS, superoxide anion, and mitochondrial superoxide production was detected as previously described through flow cytometry [98,99].

### 4.7. Detection of ΔΨm

HEK293 cells (2.5 × 10^5^ cells/mL) were cultured with 50 or 75 μM quercetin and then treated with 500 μM indomethacin for 24 h. The cells were subsequently collected and labeled for 30 min at 37 °C with 500 nM DiOC6(3). Flow cytometry was used to analyze the fluorescence intensity corresponding to ΔΨm in accordance with previously described methods [96,100,101].

### 4.8. Apoptotic mRNA Level Analysis

Cells (1 × 10^6^ cells/total) were exposed for 24 h to 500 μM indomethacin with or without 75 μM quercetin. The assay proceeded in accordance with the protocol of the Qiagen RNeasy Mini Kit as described previously [102]. RNA samples were processed with kit reagent in accordance with the manufacturer’s protocol (Applied Biosystems, Foster City, CA, USA) for 30 min at 42 °C. The following protocol was used in the subsequent quantitative polymerase chain reaction: 2 min at 50 °C, 10 min at 95 °C, 40 cycles of 15 s each at 95 °C, and 1 min at 60 °C with 1 μL of complementary DNA that was reverse-transcribed as described previously, 2X SYBR Green PCR Master Mix (Applied Biosystems), and the forward and reverse primers (200 nM; Table 1). All assays were performed three times on an Applied Biosystems 7300 Real-Time PCR System, and the comparative CT method was used to calculate the fold changes in mRNA level.

### 4.9. Statistical Analysis

Dunnett’s test and one-way analysis of variance were performed in SPSS version 16.0 (SPSS, Chicago, IL, USA). All values are the means ± standard errors of the triplicate assays. A *p* of <0.05 was considered to indicate statistical significance.

## 5. Conclusions

NSAID abuse is prevalent among athletes. The present study demonstrated that indomethacin reduces mitochondrial malfunction in HEK293 cells by affecting ROS production and ΔΨm imbalance. NSAIDs have adverse effects on the kidneys. Because no restrictions have been enacted on nonsteroidal analgesics or anti-inflammatory drugs in athletes, athletes are susceptible to NSAID abuse. Herein, we report the risk of indomethacin-induced renal malfunction; the results can provide professionals with insight for medication prescription among athletes. In addition, we identified quercetin as a natural reagent that can prevent mitochondrial malfunction and even apoptosis in indomethacin-treated HEK293 cells. The results suggest that combined quercetin and indomethacin therapy is feasible.

## Figures and Tables

**Table 1:** Primers used for apoptotic mRNA level analysis.

Primers	Sequences
homo caspase-3	5′-CAGTGGAGGCCGACTTCTTG-3′ 3′-TGGCACAAAGCGACTGGAT-5′
homo caspase-9	5′-TGTCCTACTCTACTTTCCCAGGTTTT-3′ 3′-GTGAGCCCACTGCTCAAAGAT-5′
homo AIF	5′-GGGAGGACTACGGCAAAGGT-3′ 3′-CTTCCTTGCTATTGGCATTCG-5′
homo Endo G	5′-GTACCAGGTCATCGGCAAGAA-3′ 3′-CGTAGGTGCGGAGCTCAATT-5′

## Supplementary Materials

The following are available online at https://www.mdpi.com/article/10.3390/life11111134/s1, Table S1: Supplement raw data.

## Abbreviations

ROS	Reactive oxygen species
Apaf-1	Apoptotic protease activating factor-1
AIF	Apoptosis-Inducing Factor
Fas	Fas ligand
CD95	Receptor CD95
DR4	Death receptor 4
DR5	Death receptor 5
TNFR	TNF-receptor
EndoG	Endonuclease G
EDTA	Ethylenediaminetetraacetic acid
