# Supplementary material for: Effect of Quercetin on Injury to Indomethacin-Treated Human Embryonic Kidney 293 Cells
Source: Life (Basel). 2021 Oct 25;11(11):1134. doi: 10.3390/life11111134 (PMC8623736; doi:10.3390/life11111134)
Supplement: Supplementary file 1 [file life-11-01134-s001.zip › life-1381070-SI/life-1381070-supplementary.pdf]

Table S1. Raw data.

		Cell viabillity (%)		Protection (%)	TUNEL$^+$ (%)		Caspase-3 (fold)		Caspase-9 (fold)	
		Means	SE		Means	SE	Means	SE	Means	SE
Fig. 1A, 2A, 2B	Indo 0 μM + Qct 0 μM	100.00	0.00				1.00	0.00	1.00	0.00
	Indo 125 μM + Qct 0 μM	92.92	1.53				1.19	0.08	1.05	0.04
	Indo 250μM + Qct 0 μM	83.86	2.32				1.65	0.19	1.21	0.03
	Indo 500μM + Qct 0 μM	76.68	0.74				2.15	0.15	1.77	0.20
	Indo 1000 μM + Qct 0 μM	50.28	2.05				3.26	0.17	2.70	0.45
Fig. 1B	Indo 0 μM + Qct 0 μM	100.00	0.00							
	Indo 0 μM + Qct 25μM	94.25	1.48							
	Indo 0 μM + Qct 50 μM	92.42	0.70							
	Indo 0 μM + Qct 75μM	90.42	1.22							
	Indo 0 μM + Qct 100 μM	85.28	2.61							
Fig. 1C, 1D, 2C, 2D	Indo 0 μM + Qct 0 μM	100.00	0.00		100.00	0.00	1.00	0.00	1.00	0.00
	Indo 500 μM + Qct 0μM	52.27	2.98	0.00	172.38	8.10	3.67	0.58	3.82	0.44
	Indo 500 μM + Qct 25 μM	55.27	1.06	5.74						
	Indo 500 μM + Qct 50μM	63.06	1.03	20.64	144.04	5.39	2.73	0.25	3.30	0.23
	Indo 500 μM + Qct 75 μM	74.28	2.59	42.11	124.80	4.95	2.00	0.02	2.39	0.28

Indo: indomethacin; Qct: Quercetin
